# Torsion and Bending in the Neck and Tail of Sauropod Dinosaurs and the Function of Cervical Ribs: Insights from Functional Morphology and Biomechanics

**DOI:** 10.1371/journal.pone.0078574

**Published:** 2013-10-30

**Authors:** Holger Preuschoft, Nicole Klein

**Affiliations:** 1 Anatomical Institute, Ruhr University Bochum, Bochum, Germany; 2 Staatliches Museum für Naturkunde Stuttgart, Stuttgart, Germany; Raymond M. Alf Museum of Paleontology, United States of America

## Abstract

The long necks of sauropods have been subject to many studies regarding their posture and flexibility. Length of the neck varies among groups. Here, we investigate neck posture and morphology in several clades from a mechanical viewpoint. Emphasis is put on comparing sauropod necks and tails with structures in living archosaurs and mammals. Differences in the use made of necks and tails lead to clear-cut differences in the mechanical loads occurring in the same models. Ways of sustaining loads are identified by theoretical considerations. If the observed skeletal structures are suited to resist the estimated loading in a particular posture, this concordance is taken as an argument that this posture or movement was of importance during the life of the individual. Apart from the often-discussed bending in side view, we analyze the often overlooked torsion. Because torsional stresses in a homogenous element concentrate near the periphery, a cylindrical cross section gives greatest strength, and the direction of forces is oblique. In a vertebrate neck, during e.g. shaking the head and twisting the neck, oblique muscles, like the mm. scaleni, if activated unilaterally initiate movement, counterbalance the torsional moments and keep the joints between neck vertebrae in equilibrium. If activated bilaterally, these muscles keep the neck balanced in an energy-saving upright posture. The tendons of the mm. scaleni may have ossified as cervical ribs The long cervical ribs in brachiosaurids and mamenchisaurids seem to have limited flexibility, whereas the shorter cervical ribs in Diplodocidae allowed free movement. The tails of sauropods do not show pronounced adaptation to torsion, and seem to have been carried more or less in a horizontal, extended posture. In this respect, sauropod tails resemble the necks of herbivorous cursorial mammals. These analyses provide an improved understanding of neck use that will be extended to other sauropods in subsequent studies.

## Introduction

### Neck Length and Neck Posture in Sauropod Dinosaurs

Sauropoda are a major clade of the Dinosauria (Saurischia) and the largest animals that ever lived on land, reaching body masses up to 70 tons or even more (summarized in [1], appendix). In spite of a high taxonomic diversity, all Sauropoda share a characteristic body plan, consisting of a small head, an elongated neck, a barrel-shaped trunk on four column-like limbs, and an elongated tail. The very long neck is one major hallmark of sauropod dinosaurs and may be a key innovation for their success and gigantism [2], [3]. Although all sauropods have a long neck, they show differences in neck length, morphology, and probably also in neck posture. The “morphological disparity” among sauropods was also emphasized by Taylor and Naish [4] and Taylor and Wedel [5]. In some sauropod taxa (e.g., *Mamenchisaurus*, *Omeiosaurus*) the neck was extremely long, making up approximately half of the entire body length of the animal. This was the result of an increase in the number of cervical vertebrae (up to 19) and partially also of the elongation of the single elements [6]. The necks of most Diplodocidae are not as long as in mamenchisaurids but are still elongated, with 15 to 16 cervical vertebrae. Among Sauropoda, one group, the brachiosaurids had longer forelimbs than hind limbs and are commonly thought to have kept their long necks in a more upright (vertical) posture. They have a lower segment number (around 14 cervical vertebrae) but the single cervicals are elongated. Camarasaurids, *Dicraeosaurus* and *Brachytrachelopan* are the exceptions among Sauropoda because they had rather short dimensioned necks (although the neck of camarasaurids is still elongated when compared e.g., to a giraffe), which possess unusually long and split neural processes. These forms are not considered here but will be the focus of a future study.

**Figure 1:**
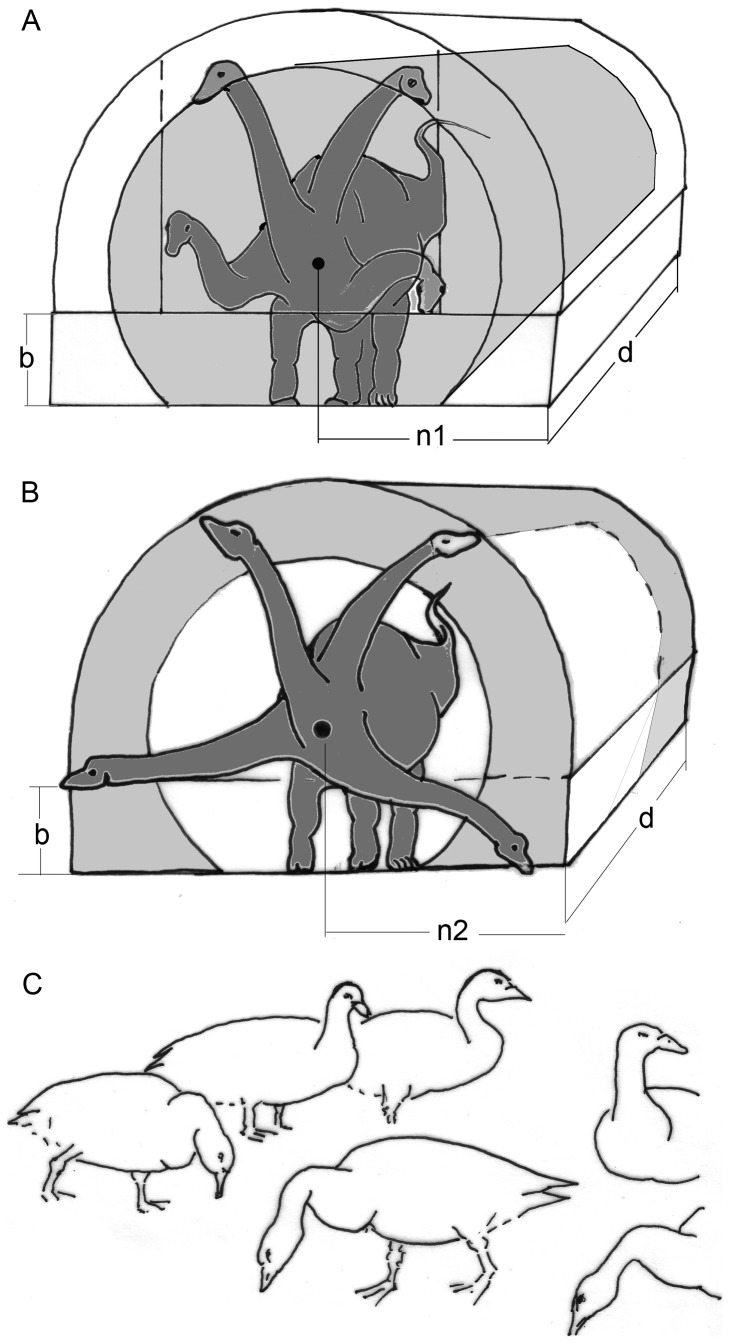
Influence of neck flexibility on the feeding envelope. A) A flexible neck with limited excursion angles allows harvesting of a sector (yellow) of the theoretically possible entire feeding envelope. B) Free excursions at the basis of an otherwise stiff and inflexible neck give access to only a peripheral part of the entire potential feeding envelope. C) Long-necked Canadian geese can and do flex their necks freely. In relaxed resting as well as in watching positions, the necks are kept upright. Both neck positions keep energy requirements low. While feeding, birds usually reduce the bending moments acting along their necks by assuming a sigmoid neck posture: near the trunk the usual downward convexity, near the head a convexity directed upward. These curvatures of the neck reduce the lever lengths, specifically the distances between the neck base and the segment weights contained in the neck. Abbreviations: b  =  forelimb length, n  =  neck length, d  =  the distance c overed during a given time. All these values are of the same size in A and B.

The most plausible explanation for the evolution of long necks in sauropods is that feeding becomes more energetically efficient by giving the animal long reaching distance for getting a hold of food without moving the entire body (e.g. [3], [7], [8], [9]). Whether this long reach is actually used for harvesting vegetation close to the ground, high in the canopy, or in any other stratum, is just a matter of the preferred food--the mechanical needs are identical. The often discussed discrimination between high and low browsing confines, in fact, the general problem to just one aspect. Only if the long necks of sauropods can be flexed in all directions is the complete exploitation of the huge volume of vegetation available for them [9]. Any restriction of neck mobility reduces the harvested volume (Fig. 1). However, strict preferences for feeding height and vegetation are common among living mammalian herbivores (e.g., giraffe [10], domestic horses, cattle, sheep, and goat [own results, data collected by Schlunk, unpublished), although all these animals are able to make use of other strata of vegetation as well. Similar preferences for feeding heights and vegetation may also have existed in sauropods.

The true neck posture in sauropods is still unknown and controversial (e.g., papers in this collection), at least in taxa such as e.g., *Giraffatitan* and mamenchisaurids. Although some authors favored nearly vertical neck postures and specialized high-browsing [11], [12], others have argued that increased horizontal feeding range has been the primary function of the neck and that the vertical range was limited [13], [14]. The flexibility of the sauropod neck is also a topic of debate. Clearly, a flexible neck allows more complete exploitation of the food resources than a restricted range of movement (Fig. 1), but the use of energy-saving tensile structures like ligaments for positioning the neck instead of energy-consuming musculature may outweigh the disadvantage of such restrictions. Standard mechanical laws were used to reconstruct the neck posture of sauropods but the results are still differing. This is partly because of different interpretations of the function of mechanically relevant structures such as the cervical ribs or the assumed amount of intervertebral and zygapophyseal cartilage (e.g. [7], [11]-[17]).

A weak point of previous approaches to neck mechanics is their bias towards bending of the neck under the influence of weight and balancing of the head and neck weight in lateral view. Several basic biomechanical facts were not given adequate attention in this context. This is particularly the occurrence of torsional loads in flexed necks that had been recognized already by Dzemski and Christian [18]. Taylor and Wedel [5] at least mention loading of the neck by lateral bending and by torsion. To improve the basic concepts of neck posture, we approach the problem here from the viewpoint of functional morphology (in a strict sense), and, literally, from different views. Essential, but also largely unknown, is the arrangement of tension-resistant structures. Since these are usually not preserved in fossils, we have to make assumptions. To narrow down the multitude of possible assumptions, we use possible homologies with crocodiles and other living vertebrates, especially birds and mammals. The biomechanical analyses will be treated as results, replacing to some extent empirical data. Our “inverse” biomechanical analysis starts off from a structure (morphology) of the skeleton, and aims to determine the (unknown) behavior. In this article, we intend to broaden the basis of analysis by including a commonly underestimated stress quality, i.e., torsion, as well as comparisons of sauropod necks with sauropod tails and non-sauropod necks. The discussion focuses on comparisons with other vertebrates, which are seen as functional analogs to sauropods.

### Cervical Ribs and Ossification of Tendons

Little attention has so far been paid to the meaning of the cervical ribs, which occur in all amniotes but are often reduced; e.g., in mammals. A typical cervical rib runs nearly parallel to the neck axis and carries an anterior and a posterior process [19]. The head of a cervical rib is divided into the dorsally located tuberculum and the ventrally located capitulum. The tuberculum connects the cervical rib dorsolaterally to the diapophysis of the neural arch and the capitulum is attached ventrolaterally to the parapophysis, which can be located on the centrum or the neural arch [19]. In sauropods, the posterior processes of cervical ribs may be shorter than, as long as its corresponding vertebra, or “hyperelongated” and extending back over several (two or more) cervical vertebrae. Such extremely long posterior processes of cervical ribs exist, for example, in *Giraffatitan brancai* and mamenchisaurids. In *Shunosaurus* and Diplodocoidea, the posterior processes of the cervical ribs are commonly shorter.

Frey and Martin [15] and Martin et al. [16] proposed a ventral bracing hypothesis in which the overlapping cervical ribs were bound into continuous rods by connective tissue and supported the neck ventrally. Following this hypothesis, cervical ribs transferred compressive forces and counteracted the torques of weight, which otherwise would have required a very muscular epaxial neck. The ventral bracing hypothesis implies a rather horizontal neck posture and is only reasonable for an inflexible neck, because any deviation from a maximum ventrally flexed and extended position would have reduced the load, and thereby the bracing function, of the rod formed by the cervical ribs. Lateral flexion would have been largely restricted [20]. Contrary to the ventral bracing hypothesis, several authors [12], [17], [21], [22] had postulated a construction which can be summarized as the “tensile member hypothesis”, in which the cervical ribs were used for transferring tensile forces over long distances, so that neck muscles could be shifted towards the trunk, thereby reducing the weight of the neck. The tensile member hypothesis is in agreement with all other tetrapod animals and allows more flexibility of the neck and is also in accordance with the dorsoventral neck mobility observed by Christian and Dzemski [20].

**Figure 2:**
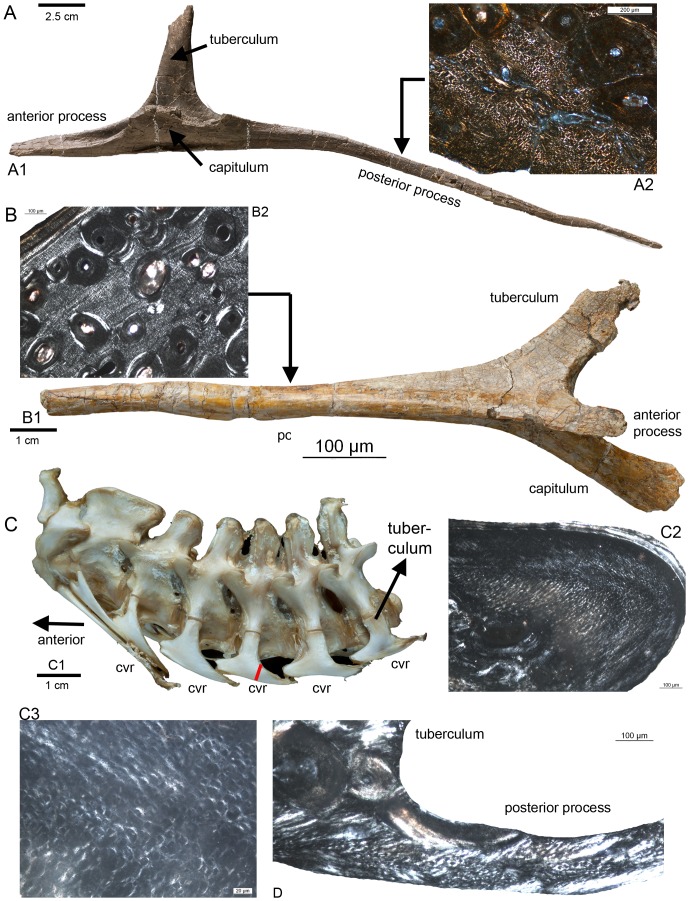
Longitudinal fibers in the posterior processes of cervical ribs of some archosaurs. A1) Cervical rib from the mid-neck region of a cf. *Diplodocus* sp. (Sauriermuseum Aathal, Aathal SMA HQ2) in ventromedial view. A2) Histological details of the posterior process of the cervical rib of a cf. *Diplodocus* sp. (SMA HQ2-D) in polarized light showing dense longitudinally running fibres between scattered secondary osteons. Note the diamond shape of the perpendicular cut longitudinal fibres. The fibres are surrounded by a sheath, which appears here mainly in white (see also Klein et al. 2012). B1) Cervical rib from the sauropodomorph *Plateosaurus engelhardti* (STIPB R 620) in ventrolateral view. B2) Histological details of the posterior process of the cervical rib of *Plateosaurus engelhardti* in polarized light showing dense longitudinally running fibers between scattered secondary osteons. C1) Neck from *Alligator missisipiensis* (STIPB R 599) in lateral view, exhibiting the cervical ribs still attached to the cervicals. In lateral view is only the dorsally located tuberculum visible. cvr  =  cervical rib. C2) Histological details of the posterior process of a mid-cervical rib of *Alligator missisipiensis* in polarized light showing dense longitudinally running fibers. C3) Enlargement of the same section, showing longitudinal running fibers. The red line on the posterior process of the mid-cervical rib marks the histological sampling location shown in C2 and C3. D) Histological sample of a posterior process of a mid-cervical rib of an ostrich (*Struthio camelus*, STIPB R 621) in lateral view and in polarized light showing longitudinally running fibers.

In two independent histological studies Cerda [23] and Klein et al. [24] found that the anterior and posterior processes of the cervical ribs of sauropods largely consist of longitudinally oriented mineralized collagen fibers, similar to what is known of the microstructure of ossified tendons [25], [24]. The tuberculum and capitulum, however, consist of periosteal compression-resistant bone [24]. The anterior and posterior processes of the cervical ribs of the alligator (*Alligator missisipiensis*), of the ostrich (*Struthio camelus*), and of the sauropodomporh *Plateosaurus engelhardi*, also show longitudinal fibers instead of periosteal bone, indicating their origin as ossified tendons (Fig. 2).

In a living organism, bone is deposited in places that are not exposed to movement ([26], [27], both resting on the experience of orthopedists and surgeons). If deformations (e.g., resulting from a fracture) cannot be excluded, ossification will not take place – so that a pseudarthrosis is developed instead of rigid bone. In addition, bone is modeled and remodeled under the influence of mechanical stress.

An old controversy is which sort of stress is responsible for the development of bone (older literature on mammals, summarized in [27]). Sverdlova and Witzel [28] recently have provided strong arguments that compressive stress alone leads to bone deposition, but this analysis was also confined to mammals. Among many birds (and possibly among sauropods and other dinosaurs) tendons have a marked tendency to ossify under the influence of tensile force (see also [29]) and in the absence of deformation. Forces on tendons are by definition produced by the muscles from which the tendons arise. Consequently, the only mechanical function known for all tendons is the transmission of tensile force, without regard to their being ossified or not. The strength of both ossified and fibrous tendons is largely the same. Therefore, the existence of an ossified tendon raises the question from which muscle it takes its origin and which function is performed by this muscle-tendon complex.

In a flexible neck, one insertion of an independent muscle is to be expected for each segment, although the transmission of tendon force on a bony element can also take place by passing a tendon through an annular ligament, which forces the tendon to change its direction or by crossing a protruding “hypomochlion”. The classical examples of such arrangements are the digits [30]-[32] and the knee joint [27], [33].

The length of the ossified part of a tendon also implies that little to no deformation took place along its length, at least during development. The length of ossified tendons also tells us how far the muscle has been removed from the insertion of its tendon, although the origin of the tendon from pinnate muscle fibers may extend far into the muscle belly. A long ossified tendon thus indicates that a tensile force was exerted again and again in exactly the same direction, and that the distance between muscle belly and insertion was long. The constant direction of pulling forces would be in agreement with the observation of a relatively stiff middle section in the necks of various animals [20]. The advantage of long tendons in slender, rapidly moved segments is convincingly explained in Klein et al. [24] by the reduction of mass along the neck and a concentration of the heavy muscle bellies in the posterior neck/anterior trunk region. The same mechanical principle has also been observed in the extremities of cursorial animals [34]-[36]. The great number of segments allows flexibility along the entire neck. Although necks of sauropods as well as those of birds are composed of many segments (i.e., cervical vertebrae), the range of movement of one segment against the next is limited [37]. Pronouncedly flexed neck postures seem to imply deformation of the cervical ribs, which would inhibit their ossification.

One major aim of the current paper is to understand what the longitudinal splitting of the ventrally flexing musculature (m. longus colli ventralis in birds) implies for neck posture and neck mobility in sauropod dinosaurs and how their cervical ribs can show such a marked tendency for elongation and ossification. We intend to develop a complete explanation for at least one among the varying shapes of necks in sauropods.

## Methods

### Premises for the Theoretical Approach

Our basic hypothesis is that all parts of the locomotor apparatus, including the neck, are optimized for fulfilling their functions that are “adapted” to sustain the loads applied during every-day life, while being as lightly built as possible with the available materials. This is in accordance with evolutionary theory and with Wolff´s law [26] and Pauwels` theory of causal morphology [27]. Both lead automatically to optimized “light-weight constructions” of the locomotor apparatus.

The most promising way to obtain better understanding of morphology is the investigation of extant vertebrates. The moments and internal forces evoked in static and kinetic conditions, as well as the skeletal and muscular structures, which resist these internal forces, can be studied directly in living forms. Because sauropods are extinct and have no living counterpart, this aim can best be approached by investigating their closest living relatives, crocodiles and birds, and functionally similar conditions in large cursorial mammals.

Crocodiles, however, do not have a very long neck, which characterizes sauropods. In addition, crocodilian tails fulfill a very special biological role, namely propelling the animal in water. Therefore, they are not fully convincing models, especially for studying the bending stresses in a long neck. The conditions of balancing the head in common terrestrial postures must be fulfilled by their morphological structures (bones, muscles and tendons) as well. Crocodiles do, however, show a behavior by which the neck is exposed to pure torsion: the often so-called “death roll” they use for hunting and feeding. As long as the animals are supported by water, no strong bending moments due to gravity obscure the torsional stressing of the neck. Special morphological traits of the crocodilés neck therefore seem to depend largely upon torsion and can be considered as “adaptations” to torsional loads. Therefore, it is reasonable to search for convergences of neck morphology between crocodiles and sauropods in spite of their obvious morphological and behavioral differences. Likewise, the tails of sauropods can be compared with those of crocodiles, although the use in each animal is different.

Obtaining data on internal forces is technically very difficult, and inflicting damage to the experimental animal is nearly inevitable. External forces can be measured with the aid of extremely expensive machinery. Muscle activities are relatively easy to monitor by using the EMG technique, but the data obtained do not tell the whole story. The most relevant shortcoming is that EMG does not yield reliable data on the forces exerted by muscles. Therefore, the simplest way to develop a reliable idea about the biomechanical conditions is a theoretical approach, which is based on standard mechanical laws.

### Our Theoretical Approach

The theoretical approach begins with precisely defined and plausible data and calculation of internal forces and stresses that show which structures are required and which shapes fit best. In fossils, the argument must be inverted: Only the skeletal structures are known, and we try to derive from them – under the premise of the basic working hypothesis of perfect “adaptation” of structures to function – the internal forces, the external loads, and finally body posture and the mode of locomotion. While the first step is quite reliable, the third and fourth steps are increasingly hypothetical – although still based on the laws of physics. Since these laws are generally valid, any agreement of traits in a living animal with identified mechanical rules confirms the correctness of the basic working hypothesis. The opposite, of course, is also true: If no agreement can be found, something must be wrong – in most cases, the error lies in the assumptions about stresses, which occur in particular movements.

The methods of theoretical mechanics are described in detail in several textbooks. Our preferred references are Lehmann [38] and Dubbel [39]. We use the terminology and common abbreviations developed by engineers. The most frequently used abbreviations are *m* for mass, *F* for force, *r* or *l* for length of the lever arm, or other distances. The product of the latter, the “moment” is named *M*. Weight is the product of mass (*m*) times Earth' acceleration (*g*).The technical models were transformed to fit the shapes of animals. Extant animals that can be observed are the most informative, with emphasis on their neck posture and mobility as well as on their locomotive behavior. During locomotion the highest forces occur and must be sustained by the animal. If this condition is not met, the resulting failure is fatal for the animal. Lower forces, which occur in social or comfort behavior, of course can be sustained by stronger structures. Extant animals also can be dissected to identify the soft-part structures like ligaments and muscles, including their insertions at the skeleton. Information about the skeleton can be derived from both extant as well as extinct forms. Fossils only reveal information about their skeletons (bones) – the aim of our work is to obtain the missing information about their “mechanical function” in the sense of Bock and v. Wahlert [40] and understand the resulting implications for the behavior of Sauropoda.

## Material

Most specimens considered in the current study are on display in public museums. All mentioned museums gave permission to study the specimens in their exhibition and/or collection.The sauropods *Brachiosaurus* (now *Giraffatitan,* Museum für Naturkunde, Berlin, Germany; MB.R.5002.1, MB.R.5002.3 - MB.R.5002.26, MB.R.5002.29, MB.R.5004, MB.R.5005.1-4 - MB.R.5007.1-19, MB.R.5000.1-25, MB.R.5000.26-50) and *Diplodocus* (e.g., *Diplodocus carnegii*, Naturmuseum Senckenberg, Frankfurt, Germany; SMF R462) were studied first hand. In addition, published illustrations of a number of other Sauropoda were considered. Our study is also based on first-hand observations and measurements of skeletons of several recent cursorial mammals in the collections of Institut d´Anatomie of the Université Louis Pasteur in Strasbourg, France, and the Naturmuseum Senckenberg, in combination with repeated observations of locomotor behavior of extant mammals. Names of the muscles follow the terminology of Fürbringer [41].

## Results

### Sauropod Necks in Different Views: Bending Under Weight

**Figure 3:**
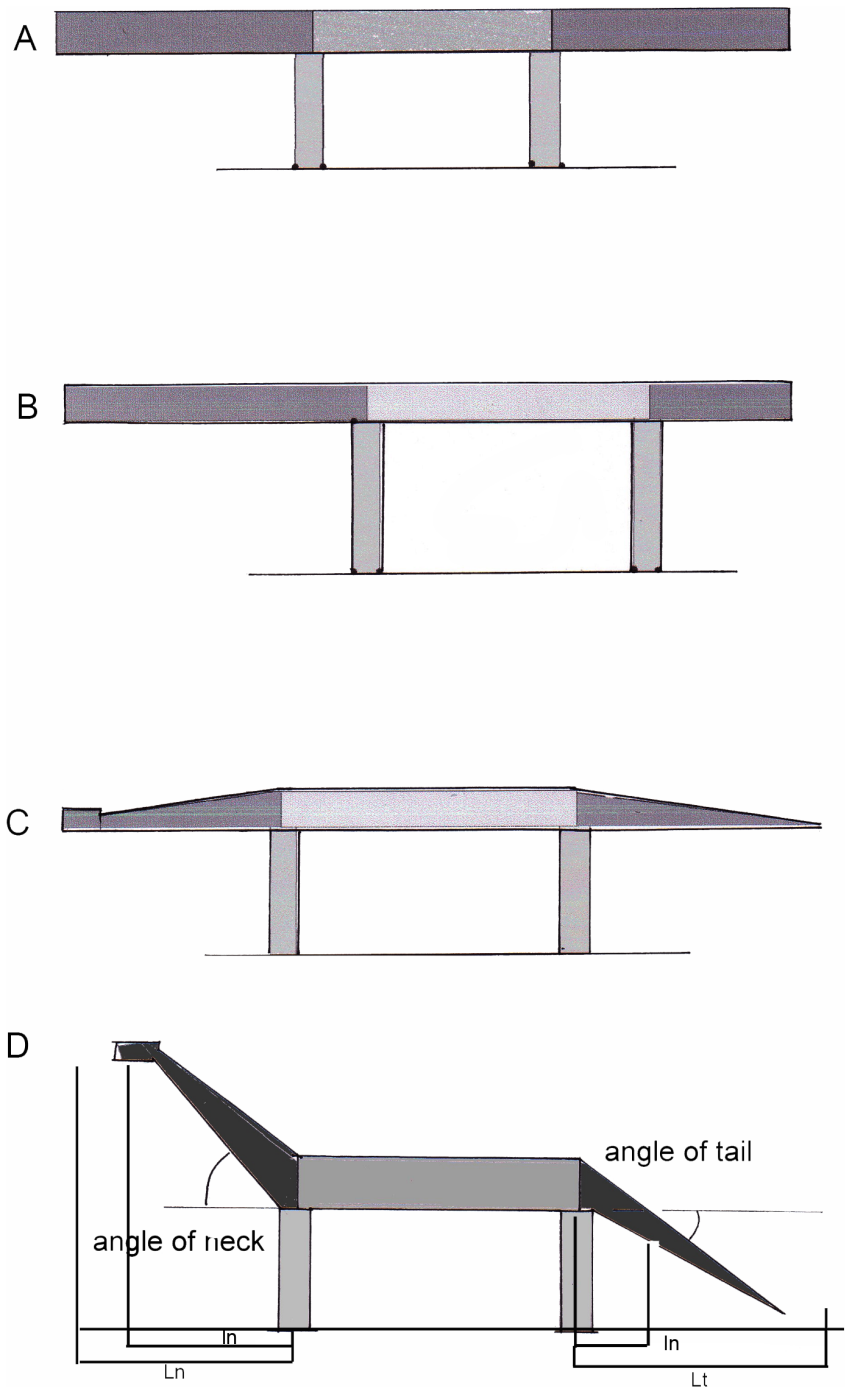
Simplified model of a sauropod dinosaur: A heavy beam on two pairs of support (limbs). The bending moments vary dependent from the lengths of the segments (A and B), dependent from the mass distribution (A and C), and dependent from the inclination of the cantilevers at both ends (A and D). The current study is focused on the cantilever segments (dark grey). L is full neck or tail length, l indicates the lever lengths of segment weights.

Seen from the side, the body of a quadrupedal animal can be compared roughly to a beam, or girder, with two cantilevers on either side, jutting out forward (head and neck) as well as rearward (tail) (Fig. 3). This construction is to be analyzed under static and under kinetic conditions. To get hold of the conditions in the three dimensions of space, the construction must be investigated from the side, top, and front. The anterior and posterior support is then, respectively, a pair of fore- and hindlimbs, and the movements –viewed from top-- of neck and tail outside the lateral plane become visible. This is not only relevant when the animal is feeding, but also during locomotion. Additional information can be supplemented by looking at the neck in the anterior or posterior views (see below). If viewed from the side, the most obvious stress quality is **bending**, evoked by the masses of the segments of the beam, or body stem, multiplied with gravity ( =  weight forces) and lever arms ( =  bending moments). The weight forces act in a vertical direction and their lever arms are greatest if the neck is kept horizontal. This leads to very high bending moments, which must be counteracted by tension-resistant structures (ligaments, muscles) on the dorsal side of the neck. The morphological “adaptations” of the sauropod neck to bending under the influence of body weight have received much attention in the literature [20], [21], [42], [43]. While the length of the neck is documented by the fossil bones, the weight of the neck is presently under discussion. Light-weight construction, especially pneumatization of the cervical vertebrae, is responsible for the very low neck densities (< 0.5) assumed by Taylor and Wedel ([5]: p16). Even if this low value is correct, the mechanical problem of controlling enormous bending moments and mass moments of inertia persists because of the extreme length of the neck. In addition, this approach does by no means explain the function of cervical ribs, which are located ventrolaterally to the column of vertebral centra; neither does the investigation of the lateral view explain the obvious morphological differences between necks and tails of dinosaurs. If viewed from the side, both are cantilevers, consisting of a big number of long rigid segments, which must be kept in equilibrium against gravity. In spite of this similarity, their shapes deviate.

Two sometimes neglected, though very simple, conditions become clearly visible in side view: first, alone muscles can keep a neck at variable heights, while the given lengths of ligaments place it in one, invariable position. Second, the absolute maximum for all moments evoked by the neck is set by the trunk mass: Neither the static moments of the neck (F_mn_ * l_n_ < F_mt_ * l_t_) nor its mass moments of inertia (F_mn_ * l_n_^2^ < F_mt_ * l_t_^2^) can become greater than that of trunk plus tail plus extremities. The same fact was noted by Taylor and Wedel [5].

**Figure 4:**
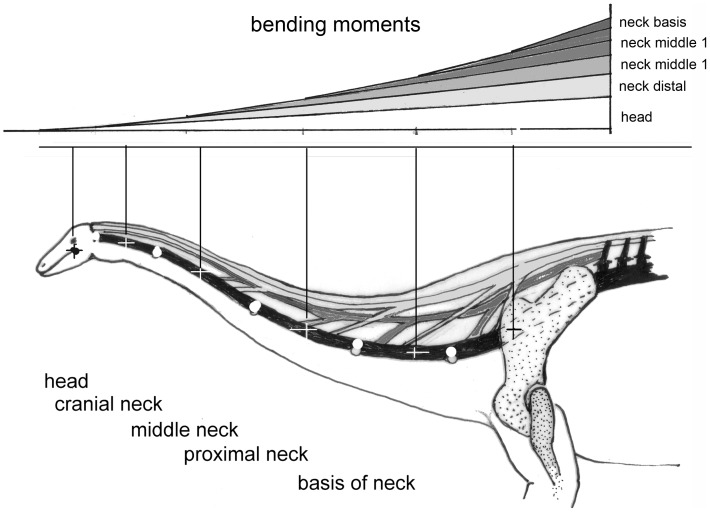
A) Schematic neck of a sauropod to show joints (open circles), centers of segment masses (crosses). B resulting bending moments. Skeletal structures are in black, ligaments are in dark grey and muscular structures are in light grey. The pull of these structures exerts compressive forces in the vertebral column (black). Note that muscular structures of variable lengths are needed to keep the joints in balance against segment weights, in all positions in which the ligaments are not pre-stretched. Stretching of the ligaments leads to forces which make further movement impossible, so setting limits to neck mobility.

The greatest forces are evoked by segment weights and by the inertia of their masses. The masses of head and neck – or of the tail – are distributed along the length. If cut into segments, each of the segments exerts a weight force (F_mi_). These forces are multiplied with their distances from the pivot to yield ventrally directed bending moments (Fm_i_ * l_i_). The bending moments add up to a maximal value at the pivot that is the joint between the most proximal neck vertebra and the most cranial thoracic vertebra (Fig. 4). The envelope of all these bending moments, exerted by all segments, follows an exponential curve (Fig. 4). The moments can be reduced by choosing flexed postures of the neck (Fig. 1), which reduce the lever arms. The bending moments evoked by weight in all postures must be balanced at each intervertebral joint by the moments exerted by one of the tension-resistant structures (muscles or ligaments). To allow free choice of postures, these structures must insert into each individual vertebra. If muscles (tension-resistant structures of variable lengths) are present, the positions of all segments can be chosen arbitrarily. This is not the case if ligaments (tension-resistant structures of defined lengths) counteract weight, because tough, collagenous ligaments have a length limit that cannot be exceeded and arrests further movement. If elastic ligaments are present, the force they produce depends upon the degree to which they are stretched. If such a ligament is not pre-stretched, it produces no or a very small force. If a given length is reached by stretching the elastic ligament, an equilibrium results and the elastic ligament stops further movement as long as the moving force (e.g. weight) remains unchanged. On the other hand, if the movement leads to a relaxation of the elastic ligament, it does not exert any force on the skeletal structures, which then can be arranged in any position, without being influenced by the ligament.

The height, to which the head is lifted on the neck, influences the bending moments by reducing the lever arms (*l*), following the formula: 1  =  L * cosine α, where L is neck length and α the angle against the horizontal (Fig. 3A).

**Figure 5:**
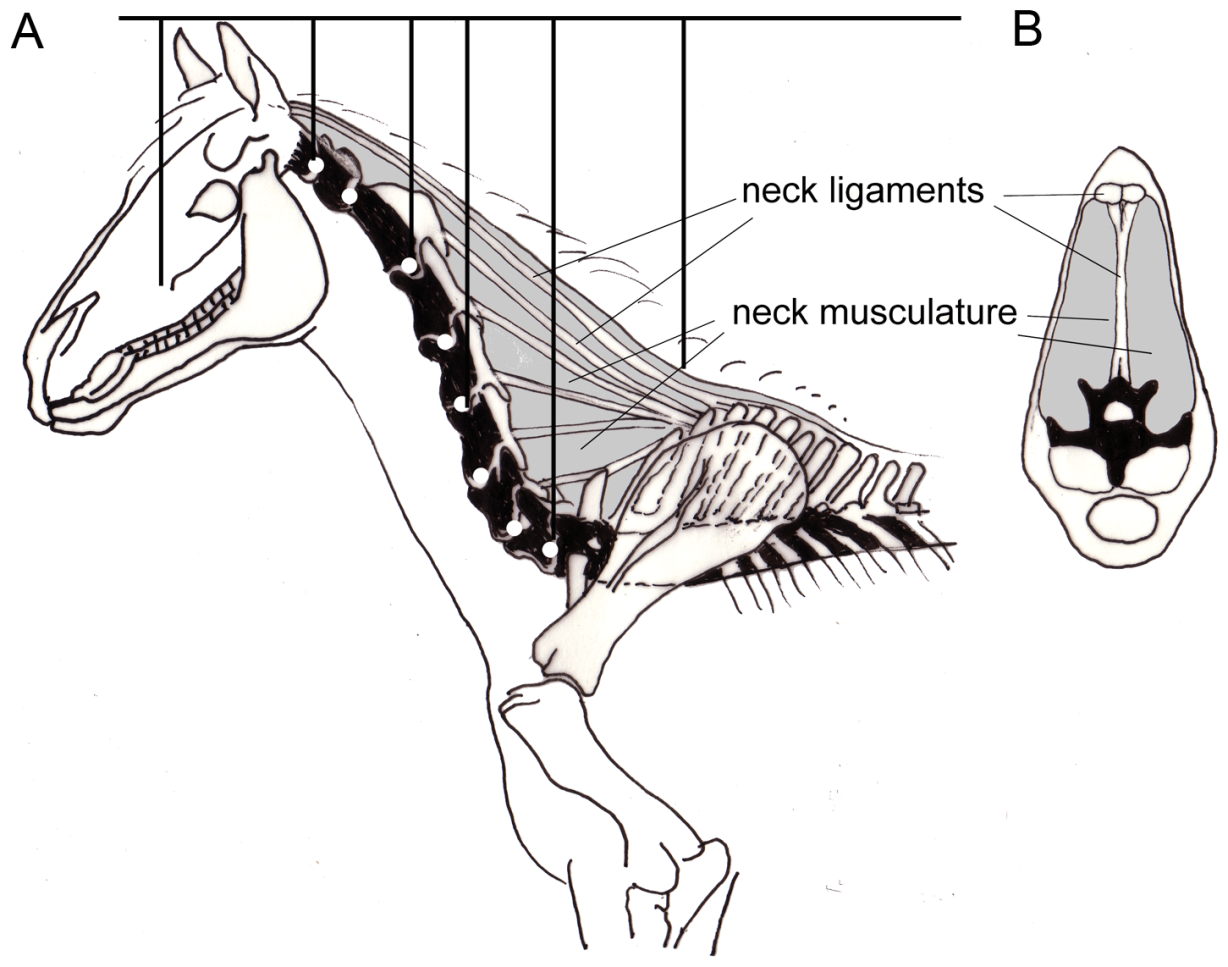
Neck of a horse as example of a cursorial mammal. A) Horse neck plus head in side view. B) Bending moments caused by segment weights in analogy to the sauropod in Figure 4. C) Cross section through a horse neck at the level of cervical 7. This arrangement of structures is highly specialized to sustain the bending moments that occur in the mediosagittal plane and are visible in side view.

**Figure 6:**
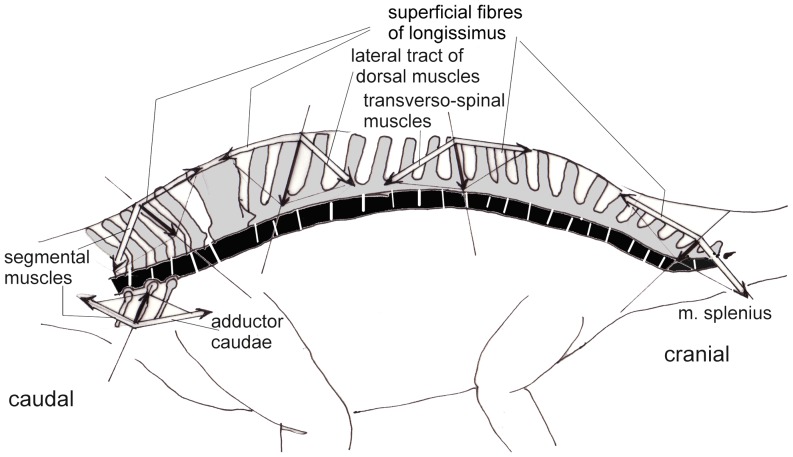
Direction and loading of spinal processes (neuroapophyses). The spinal processes of the tail of a sauropod are not exposed to bending if directed along the resultant of all forces acting on them. Instead of the ligamentum superspinale, a longitudinal muscle leads to the same result. If the muscle forces are increased, or the ligaments heavily prestretched, the resultants may well deviate at least temporarily from the spinal processes. In these cases, bending strength is required.

The necks of mammals and the tails of sauropods are also characterized by tension-resistant fibrous or muscular structures that in side view space apart from the vertebral column so that they have long lever arms, which are less pronounced in transverse direction. The farther removed from the vertebral bodies, the longer the lever arms and the greater their moments around the joints between the centra. In the neck vertebrae of sauropods and of most mammals, there are no or at least no apparent dorsal extensions of the skeletal elements ( =  spinal processes), the whole space between the vertebrae and the contour being filled with tension-resistant soft tissues. The lever arms are long because the nuchal ligaments and the muscles have their origins at the spinal processes of the anterior dorsal vertebrae, between the transverse processes (and ribs) and their tips. The reverse is true in the tails. The spinal processes are elongated in the anterior thoracal segments, forming something like a “withers” (Fig. 5A). Although only a part of the muscle fibers is attached to the spinal processes of these trunk vertebrae, the major part of the nuchal ligament in the neck or the supraspinal ligament in the tail is attached to the tips of the long spinal processes. To better resist the high longitudinal tensile force exerted by the ligaments (Figs. 4, 5A) the spinal processes of the anterior thorax are inclined rearward. By this inclination the spinal processes have the same direction as the resultant of all major forces acting on them and bending moments do not occur while the spinal processes are under compression (Fig. 6). If, however, the muscle forces are increased, or the ligaments heavily pre-streched, the resultants may deviate temporarily from the spinal processes, so that bending moments are evoked. In addition to their inclination, the spinal processes possess very large diameters in the direction of the tensile forces and therefore remarkable bending strength. It can be assumed, that this sort of loading is only transitory, because a new equilibrium is established rapidly by muscle reflexes.

### Sauropod Necks: Twisting and Torsional Stress

**Figure 7:**
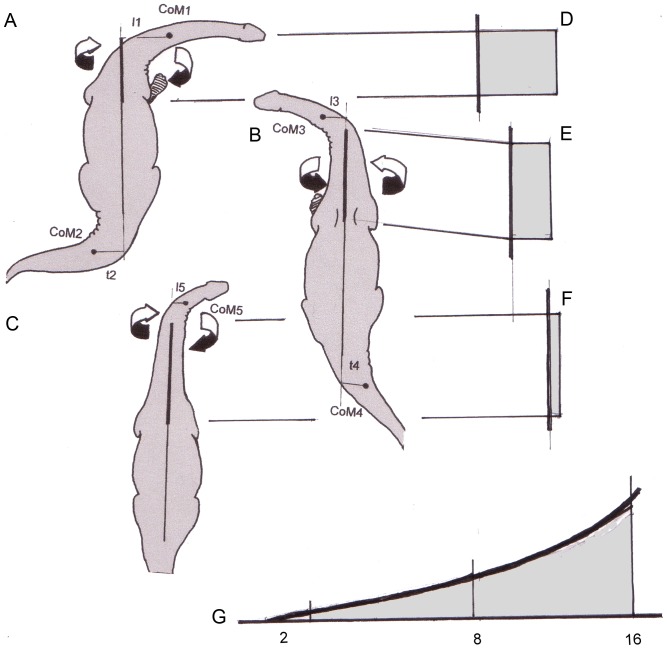
View from above (top) on sauropods, which flex their necks laterally. A – C) The proximal neck segments exposed to torsion are marked by a heavy long axis. The dots are the CoM (centers of mass) of the head plus neck segments distal to the flexed joints. The masses concentrated in the CoM (CoM 1, 3 or 5, respectively) become smaller with a distal shift of the flexure and their lever arms (l_1_ in comparison to l_3_ or l_5_) become shorter. A) The moment of the heavy and long neck is so great, that the inner (right) foot must be placed laterally in order to expand the area of support and to prevent imbalance of the whole animal. B) The same is shown for flexion of the neck to the left. C) The rotating moment is so small that it does not require a lateral placement of a forefoot. D – F) Torsional moments evoked by lateral flexion remain constant along the posterior part of the neck. In all cases shown here, the tails are flexed into the direction opposite to the neck. So the imbalance caused by lateral flexion of the neck can be reduced. The degree to which the tail can be used to counterbalance the neck depends from the ratio CoM 1 * l_1_/CoM2 * l_2_, or CoM3 * l_3_/CoM4 * l_4_, respectively. G) Maximal torsional moments that can occur along the neck from segment 2 – segment 16.

**Figure 8:**
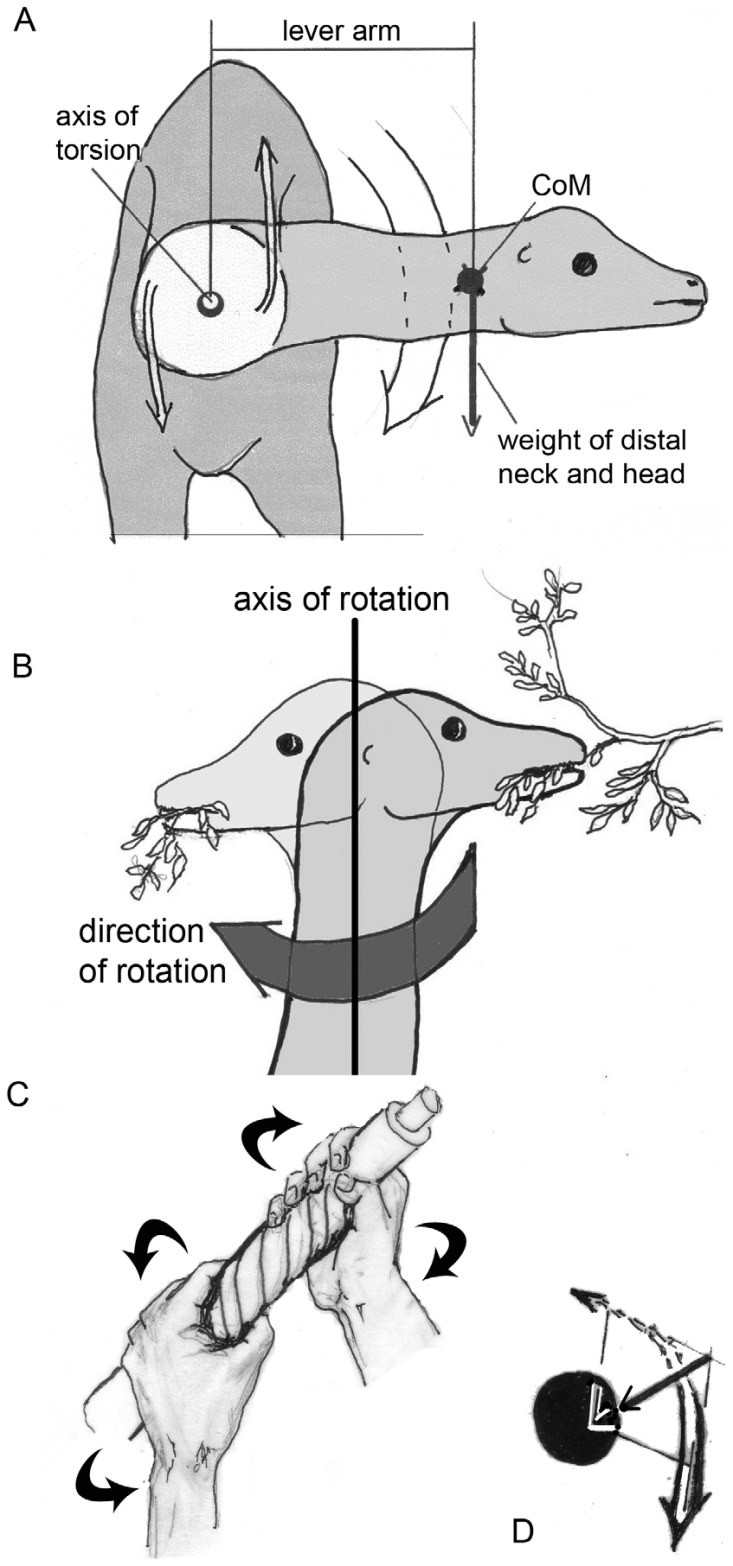
Torsional stresses which are evoked by twisting the neck. A) Anterior view on a sauropod flexing its neck laterally. The product of weight of neck plus head * lever arm is the torsional moment around the axis of rotation along the proximal part of the neck. F_m1_ and F_m2_ are schematic representations of the oblique neck muscles described in the text, whereby the components along the length of the neck are ignored. B) Vertical neck position and turning the head from right to left, e.g., for stripping foliage from a branch. The movement is resisted by the strength of the branch, which exerts a force directed to the right. This resistance must be overcome by active torsion of the distal neck. C) To realize torsion, imagine a wet cloth, which is wrung out by both hands. Its fibers form a spiral between the points of force application. Doing so, the length of the cloth between the hands is shortened, and at the same time the oblique fibers exert a resultant force against the center – exactly as the oblique muscles of the neck exert a re-directional force against the vertebral column (stick), which is also compressed in its longitudinal direction.

Additional information can be supplemented by looking at the neck in anterior or dorsal views. Figures 7 and 8A, B show hypothetical sauropods with their necks flexed laterally and Figures 8B, C show their head turned. The neck segments proximal to the lateral flexure are exposed to torsion. The torsional moments (*M_t_*) are defined by M_t_  =  F * r, where *F* is the weight of the segments distal to the bend, and r the distance from the longitudinal axis of the proximal neck to the center of mass (CoM) of the laterally flexed segments. Figures 7A and 7C illustrate muscles that counteract the torsional moments and thus give the neck the characteristics of a cantilever supported solely at its base. Actual rotation about the longitudinal axis of the neck – which is impossible in extant animals [13], [35] – is not necessary for evoking torsion. Along the twisted proximal part of the neck, torsional moments are constant (Fig. 7A-C). If, however, the lateral curvature of the neck is shifted proximally, the maximal torsional moments can increase with the masses of the neck segments distal to the curvature and with their lengths, which means the increase follows an exponential function (Fig. 7G).

**Figure 9:**
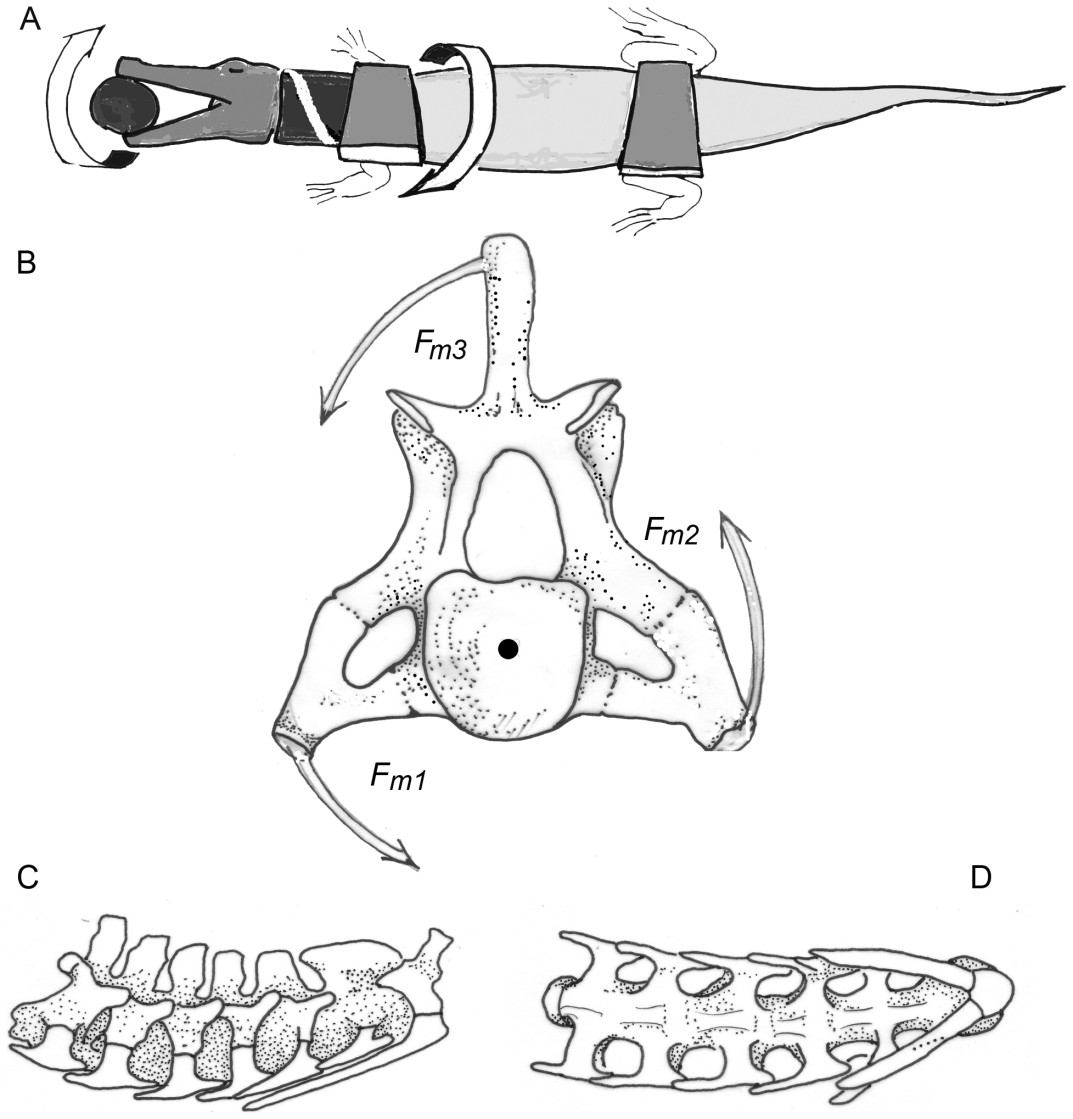
Death roll in a crocodile and its mechanical consequences for the neck. A) Schematic drawing of a crocodile performing the death roll. The head is anchored by the jaws, while the trunk rotates about its longitudinal axis. The neck segments are kept in equilibrium by muscles pulling in the direction of the white band. B) The seventh neck vertebra of an *Alligator missisipiensis* in posterior view. F_m1_-F_m3_ are the muscle components in the transverse plane which counter the torsional moments exerted while performing the death roll. C) Part of the neck skeleton of the *Alligator missisipiensis* with the cervical ribs (white) in side view. D) Part of the neck skeleton of the alligator in ventral view. The head points to the right in both cases.

The sort of loading which causes “torsion” (Figs. 7, 8) in the neck can best be illustrated and analyzed in crocodiles (Fig. 9). During the "death roll behavior", the neck is exposed to huge torsional moments when transmitting the twisting forces from the trunk to the head [44] (Fig. 9A). To keep the intervertebral joints in balance, tensile forces running obliquely from the shoulder girdle and thorax to the head must be countered by oblique muscles, which control the position of each vertebra. These muscles are arranged in chains, and some of them act directly on the head, without contact to the neck (like the m. collosquamosus and m. longissimus capitis [45]. In a crocodile, the necessary force components are labeled F_m1_, _Fm2_, and F_m3_ (Fig. 9B). Each component is provided by a group of muscles that block rotation of the neck around its longitudinal axis on the side where the head is forced to rotate dorsally and the trunk ventrally (own dissections, as well as [41], [45], [46]). The force component F_m3_ comprises the m. trapezius (connecting the lateral surface of the scapula with the spinal processes), m. rhomboideus (connecting the vertebral margin of the scapula with the spinal processes), and at the neck segments two to four, deep layers of the m. cervicis ( =  m. multifidus, extending from transverse processes of more caudal vertebrae to spinal processes of more cranial ones). Group F_m2_ includes the m. levator scapulae or m. serratus profundus (running from the vertebral margin of the scapula to the tranverse processes of the vertebrae) and, at the posterior neck segments, the m. multifidus. Component F_m1_ is provided by the m. scalenus (or m. costocervicalis, see [45]), which connects the most anterior thoracal ribs to the “neck ribs”, exerting force along their greatest length [44]. Thus, the tendons of the m. scalenus are the most probable candidates for ossification in the case of the sauropods.

In all tetrapods, the ventrally directed tensile forces are taken over from the transverse processes of the neck vertebrae by the fibers of the transversospinal or multifidus system. The muscle chain is continued on the contralateral side and the oblique tensile force is transmitted to the head by the m. splenius and m. semispinalis capitis (Fm3). The m. sternomastoideus has the same function but crosses the whole distance from sternum to head without contact to the neck vertebrae.

**Figure 10:**
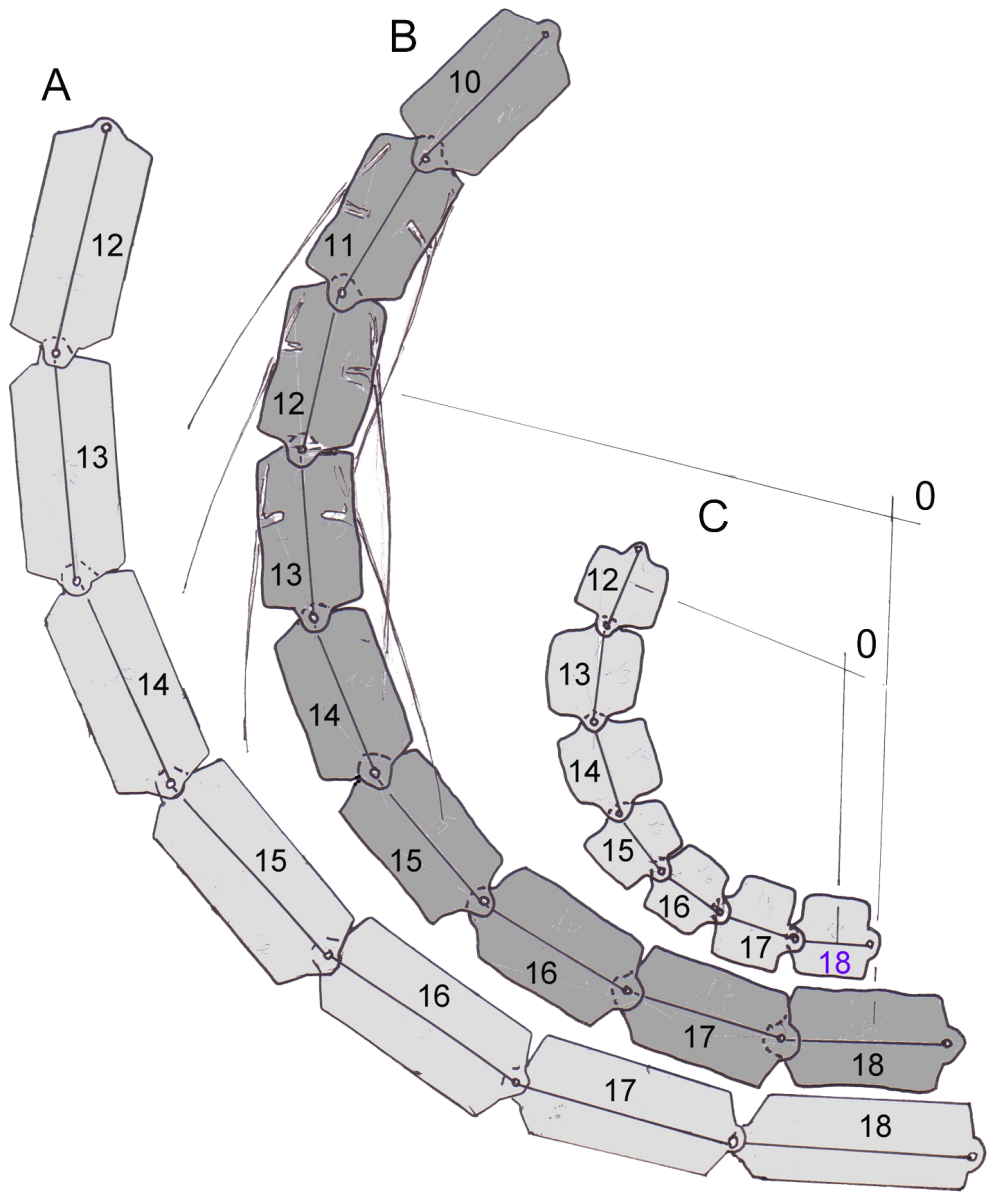
Proximal part of a schematic neck seen from on top illustrates flexibility. A) Elongation of the segments (cervical vertebrae) makes the radius of curvature longer. Note that the cervical ribs do not contact vertebrae because they deviate ventrally from the axes of the centra. B) The segments (cervical vertebrae) 12 – 18 are deflected by 20° each. This corresponds to the lateral deflection observed by Dzemski (2006) in the ostrich. In addition, (long) cervical ribs are shown on both sides of the vertebrae. C) Shortening of the segments leads to a sharper curvature of the neck.

The parapophysis of the vertebra connect the capitulum of the cervical ribs to the axial skeleton, and the diapophysis of the vertebra connects the tuberculum of the cervical ribs to the axial skeleton, and thus both keep the twisting forces far away from the neck´s longitudinal axis. Their long lever arms (Figs. 8, 9) allow reduction of muscle forces but make movements slower than short lever arms would do. By being stretched passively or by active contraction, the above mentioned muscles exert compressive forces directed medially against the axial skeleton (Fig. 10C), specifically against the capitulum and tuberculum of the cervical rib as well as against the parapophysis and diapophysis of the vertebrae. Indeed, the connections between vertebrae and cervical ribs are suited to sustain compression [24]. Because the neck skeletons of crocodiles possess morphological features very similar to those that can be observed in diplodocid and brachiosaurid sauropods, the arrangement of cervical ribs and their anchoring to the neck vertebrae can be identified as “adaptations”, well suited to resisting the torsional stresses with a minimum of material.

### Ossified Tendons on the Ventrolateral Side of the Neck and Their Implications for Neck Mobility

The long posterior processes of the cervical ribs of sauropods contain mostly longitudinal fibers [23], [24], and therefore must be interpreted as ossified tendons. This raises two questions: first, how are tension-resistant structures or muscle-tendon complexes on the ventrolateral sides of cervical centra used, and second, to which muscle(s) could they have belonged? The answer to the first question seems to be simple: If contracted on both sides simultaneously, the muscle-tendon complexes flex the neck ventrally (like the m. longus colli ventralis in birds). For the development of muscle-tendon complexes it is unimportant whether intervertebral flexion takes place or not; the tensile forces are also required for keeping balance between the segments in static situations, not just for movement. The splitting of the tendons to insert into each vertebra allows precise control of the exact position of these vertebrae.

Not so easy to answer is the question why muscles are recruited for ventral flexion in a forwardly inclined neck of a sauropod, because the weight of neck segments and the enormous distances from the segments to the base of the neck yield ventrally bending moments without any expenditure of energy – as was discussed often in the literature (e.g. [18], [37], [42], [47]) and is illustrated in Figures 5, 6, and 7. Unilateral contraction of muscles ventral to the vertebral column (Fm3 in Fig. 9B) primarily leads to ventrolateral bending and turning the distal neck segments or the head. Even if no movement takes place, stresses occur and must be counterbalanced. If the head is kept in its position, by its weight or inertia, or by foliage resisting being cropped, the torsional stresses occurring in the neck can be taken over by the muscle of only one side.

The second question raised above, to which muscle the ossified tendons belonged, is difficult to answer because soft part anatomy is not preserved and the functional analogs are contradictory: In crocodiles as well as in sauropods, the posterior processes deviate from the vertebrae caudally, which is visible in dorsal and in lateral view. They point towards the direction of a rather lateral origin, perhaps on the anterior ribs like the m. scalenus (Fm3 in Fig. 9B). From a phylogenetic perspective, it is equally likely that the muscle attaching to the cervical ribs in sauropods is the m. scalenus as in crocodiles, rather than the m. longissimus colli as in birds (see below). It should be noted that the m. longus colli passing along the ventral surfaces of the cervical centra is present in crocodiles – similar to the arrangement in mammals [45].

### Flexibility of Sauropod Necks

Sauropod necks are divided into 14 to 19 neck segments, which can be moved against each other. This is similar to the situation in birds, which has been investigated systematically by Dzemski ([37], but see also [18], [20]). The excursions in the intervertebral joints add up to the full mobility of the neck (Fig. 10). If the mobility between neighboring vertebrae as observed in ostriches is taken as an example, a total flexion of the neck by 90° is reached or exceeded by a chain of seven (proximal) segments. A greater number of segments would lead to more pronounced flexion. The degree of flexion or the “sharpness” (minimal radius) of the curvature also depends on the lengths of segments: The shorter the segments, the shorter the radius of curvature (Fig. 10C). Longer segments in turn lead to a less pronounced curvature (Fig. 10A, B). The same drawing (Fig. 10B) illustrates a side-effect of ossified tendons: if the posterior processes of the cervical ribs are shorter than or as long as the vertebrae (such as in *Diplodocus, Apatosaurus,* and *Alligator*), these rigid elements do not at all influence flexibility of the neck. At the level of the intervertebral junctions, the tendons consist of collagenous fibers, which can easily be deflected. If the posterior processes of the cervical ribs are longer than one segment (such as in *Giraffatitan* and mamenchisaurids), they approach the neck vertebral column on the concave side, while being removed from the vertebrae on the convex side of the flexion. If the posterior processes are ossified tendons, the lever arms of these tendons at the concave side of neck flexion (no matter in which plane it occurs, mediolateral or dorsoventral) are long at the nearest intervertebral joint, but decrease posteriorly until they make contact with the vertebral column. At the convex side of the curvature, the lever arms of the pulling forces increase continuously in the posterior direction, making the effect of a contraction stronger and stronger. At the posterior tip of the cervical rib, the tendon must be tied to the vertebra, either by muscular fibers attached to the bony element or by tough connective tissue, which redirects the direction of pulling force. No histological evidence exists for such a tying, however [24]. In addition, the tendons cannot escape being bent – which would make ossification improbable. Thus, only one conclusion seems convincing: that active flexibility was limited where the long ossified tendons exist. Ossification can be taken as an indicator of a straight flow of forces across several joints without any change of direction.

### Tails of Sauropods and Crocodilians

In the case of sauropods, no realistic idea seems to exist as to what the animals did with their tails – except keeping them horizontally and serving as the posterior insertion of the caudofemoralis muscle [48] or as a counterweight for the neck [49], [50]. For comparison, we consider the tails of crocodilians that serve a clear-cut function, propulsion in water. Anyway, a major functional difference between necks and tails is the application of external forces. This difference has several reasons: In spite of the lack of a masticatory apparatus as in mammals, the heads in sauropods are usually heavier than the tips of their tails. External forces are often concentrated at the jaws, while external forces acting against the tail are usually distributed over a considerable part or its entire length (for example water resistance in swimming, ground reaction forces compensating a part of the weight). Twisting plays a lesser role for the tails than for the necks. In addition, flexibility of the tail is less pronounced and the musculature is less differentiated than that of the neck in all land-living vertebrates [45], [51], [52].

Like the necks, the long tails of reptiles are exposed to bending moments because of their weight, especially if carried without ground contact more or less horizontally.

In the case of most dinosaurs no traces of drag marks of the tail have ever been found, so we may assume that this appendage was kept in a rather invariable position at the level of the pelvis, well above the ground. In view of their limited mobility, a “specialized” profile, as is characteristic for the necks of mammals (see also Fig. 5) can be expected in the tails of all sauropods. The arrangement of tension-sustaining elements with long dorsal lever arms leads to high and narrow cross sections of the tails – like in the necks of mammals (see below) and the tails of crocodiles. The arrangement of the sagittal vertebral processes, especially the presence of the neural spines on the dorsal side, limits pronounced dorsal and ventral flexion, which is in strong contrast to the situation in the neck. These processes provide firm insertions for ligaments and so reduce the need to control the taiĺs position actively by expending muscular energy.

The tail vertebrae in sauropods as well as in crocodiles also possess ventral processes (hemapophyses) of variable length in addition to the long dorsal neural spines. In *Diplodocus*, for example, the caudal vertebrae posterior to the second caudal show hemapophyses. In all cases, the “strong”, that is broad and rearward inclined, dorsal processes are rigidly fused to the neural arches, whereas the more slender ventral processes are weaker and not rigidly fused to the centra, but attached to them in a sort of joint. Although only a part of the muscle fibers is attached to the spinal processes, the major part of the supraspinal ligament is fixed to the tips of the long spinal processes. The exact direction of the resultant of all tensile forces acting on the spinal processes is not predictable. Therefore, height resistance of the rigid dorsal processes makes sense. Their inclination keeps bending moments within the neural processes and at their bases (Fig. 6) at a low level. In addition to their inclination, the spinal processes possess a shape that provides great bending strength. Obviously, the dorsal processes are better suited to sustain the bending moments exerted by tensile structures attached to their tips than the ventral processes. The rearward inclination of the dorsal processes of the tail vertebrae corresponds to the forward directed tensile force acting on them. The anterior insertions of these tensile structures are the spinal processes of the posterior trunk, which are long and inclined somewhat forward, comparable to the withers at the base of the neck in the anterior trunk segments of cursorial mammals. The combination of rigid and long dorsal processes with ligamentous passive elements of defined lengths implies rigidity, or at least limited mobility of the tail. The bending moments occurring in the necks and tails also influence the bending of the trunk region [42], [43].

If indeed the position of the tail was invariable, the tensile structures to support the tail may possess a definite length, like ligaments, rather than being able to adapt their lengths, like muscles. In fact, some dinosaurs, such as hadrosaurs, show ossified structures in their skeleton comparable to the muscle insertions in Figure 6. The advantages of ligaments and tendons are their ability to exert force (some 1700 N/cm^2^) without expenditure of energy and their lower weight. Muscles, by contrast, are weaker (something like 50 N/cm^2^, the values vary tremendously), much heavier and require much energy if actively contracted. The musculature of sauropod tails commonly is reconstructed on the basis of homology, or as in this study, of (hypothetical!) biomechanical needs. Homology can be based on the muscles of crocodiles. Unfortunately, the information in the literature is not precise, but confined to generalized statements about the stem musculature. Often authors classify muscles just as “primitive”, meaning that muscles are segmented and arranged similar to fish tails.

As noted above, the hemapophyses of the tail vertebrae are attached to the centra by joints, which provide long lever arms for the hypaxial muscles while permitting longitudinal flexion of the skeletal elements against the body axis instead of sustaining torque. In the transverse direction, the two branches forming the hemal arch provide remarkable strength against being bent in the transverse direction. The increase of strength follows a parabolic function, corresponding to loads distributed over the whole length of the hemapophyses [53]. This makes sense in the case of crocodiles, which expose their tails to water resistance, but not in sauropods. Keeping the tail in its position requires not just strong torques in the dorsal direction by the tensile structures attached to the neural spines, but also devices for keeping balance in the opposite, ventral direction. Muscles on the ventral side may well be weaker than the epaxial muscles, and indeed many dinosaurs possess shorter and more slender hemapophyses. As in the necks of sauropods, torsional stresses must be expected in long tails as soon as a lateral flexion takes place (Fig. 10). The arrangement of the m. caudofemoralis in fact is suited to control the torsion on the convex side of any lateral flexion.

In the absence of direct evidence for tail use and tail function in sauropods, it seems worthwhile to go into details of tail anatomy and function in their closest extant relatives. Crocodiles and also lizards usually tow their tails behind when walking on the ground, and lift it only in rare cases and for a short time off the ground. Bending and torsional moments are reduced by this behavior. In crocodiles that walk rapidly (“high walk”) or run on firm ground, the tail is partly balanced by the dorsal muscles, while during the “low walk” or slithering, the tail is propped against the ground or resting on it and gives the caudofemoralis muscle a solid, immobile caudal insertion for retroverting the hind limbs [54].

According to Gatesy [48], Fechner [55] and Mallison [50], the m. caudofemoralis was also responsible for retracting the hind limb during terrestrial locomotion of sauropods, just as in crocodiles. Because it connects the femur (fourth trochanter, see e.g., [56]) with the transverse processes of the anterior caudal vertebrae, the m. caudofemoralis exerts a ventrally flexing moment on the tail. Because no tail drag marks are known, ventral flexion of the tail during retraction of the thigh seems to be excluded in terrestrial sauropods by the strong tension-producing structures, muscles or ligaments, dorsal to the axial skeleton (see above).

### External Ground-Reaction Forces depending on Neck Posture

Because of its length, neck posture exerts a strong influence on the **external equilibrium** of the whole animal. The external equilibrium requires that 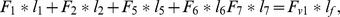



if the neck is kept horizontally, or, if the neck is elevated, 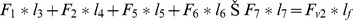



(under the condition of equilibrium of moments: sum of all moments acting on the hind foot  =  0).

**Figure 11:**
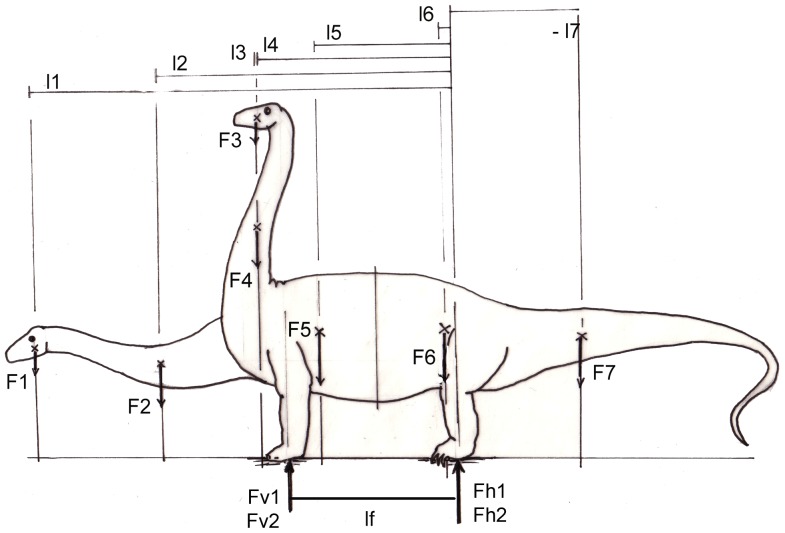
External equilibrium of a sauropod, depending on neck posture. External equilibrium is determined by the moments of segment weights about the hind feet, which must be equal to the ground reaction force F_v1_ or F_v2_, respectively, exerted by the forefeet with a lever arm l_f_ (F_v_ * l_f_). Note that the tail exerts a nose up-rotating torque, because of its negative lever arm (1_7_) Low neck position gives the weight forces of head and neck (F_1_, F_2_) long lever arms (1_1_, 1_2_). By contrast, a high neck position entails shorter lever arms (1_3_, 1_4_) of the same weight forces as before (F_1_ and F_2_). This reduces the load on the forefeet: F_v2_ in comparison to F_v1_. The share of body weight carried by the hindlimbs (F_h1_ or F_h2_, respectively, is total body weight – F_v_ The elevation of the neck is equivalent to a shift of the CoM in dorsal and caudal direction.

From the condition of equilibrium of forces (sum of all forces  =  0) follows that the sum of all segment weights (*F*) equals the sum of reaction forces (F_v_ and F_h_ in Fig. 11). Obviously, the values F_1_^*^ l_1_ + F_2_*l_2_ are greater than F_1_*l_3_ + F_2_*l_4_, and therefore the horizontally held neck leads to much higher ground reaction forces in the forelimbs (F_v1_), than a vertically erected neck (F_v2_). In other words, the center of total body mass is shifted forward by stretching the neck horizontally, and shifted rearward by assuming a more upright position. If a mass distribution similar to Henderson [57] is taken as an example, the forelimbs carry 39% of total body weight when the neck is kept horizontally, but only 26% when the neck is elevated (Fig. 11). The difference between the reaction forces on the forelimb is 33.3%. Indeed the imprints of forelimbs in the majority of sauropod tracks cover much smaller areas than those of hind limbs (Laebe, unpublished data; [58]). This clearly indicates smaller loads on the forelimbs than on the hind limbs because the ground reaction forces are distributed over the contact area (that is the sole) of the limb. In addition, the imprints of the forefeet are shallower than those of the hind feet [58]–[60], which also indicates more weight on the hind limbs, although this relationship cannot yet be quantified. Especially, lateral flexion of the horizontal neck leads not only to torsional stresses at the base of the neck, but also requires lateral placements of a forefoot including abduction in the shoulder joint (Fig. 7, 10; [61]). Such a sprawling posture would be in line with the characteristic shape of the head of the humerus and the orientation of its greatest diameter transverse to the sagittal plane in sauropods.

## Discussion

### Sauropoda

What we can see in fossils are morphological peculiarities (characters) of the skeleton, but what is missing is the functional meaning of these characters. Nevertheless, it is quite usual to talk about “adaptations”, without considering that the use of this word implies a functional hypothesis. These hypotheses are often not justified. Only if the functional value of a character can be defined clearly (perhaps quantitatively!), we may conclude that the characters are “adapted”. According to Wolff [26] and Pauwels [27], the existing shapes are developed under the influence of mechanical stresses and therefore fit perfectly to the loads acting on them: morphology is per se “adapted” and this means that the patterns of stresses under which the shape was formed can be observed. The problem we are still facing is: What makes up the relevant functional stresses, which have shaped a particular (morphological) character? The variables we are searching for by applying inverse biomechanics are body posture and mode of locomotion. Limits (or “constraints”) of our search are given by functional analogs among mammals and recent birds.

Regardless of their preferred inclination, sauropod necks are exposed to bending. The lengths of the lever arms may vary, following the cosine of the inclination angles. Sauropod necks are also exposed to considerable torsional moments. In spite of light-weight-constructions [5], [62], the enormous lengths of some sauropod necks evoke very high bending and torsional moments, especially in more or less horizontal neck postures. Counteracting against these moments requires muscle activity. The activity of muscles costs much energy, even if only slowly contracting “red” muscle fibers are involved. The fact that the lateral flexion of a more or less horizontal neck inevitably leads to torsional moments in the neck section proximal to the flexure is commonly overlooked. Our analysis shows that the structures summarized under “cervical ribs” are well suited to sustaining torsional stresses.

Cervical ribs of less than a vertebrás length remain straight and are without influence on neck flexibility even in pronounced bending of the neck (Fig. 11). Great length of the cervical ribs requires deforming the bony elements, which inhibits the process of ossification in the tendons. A general condition for ossification is the absence of any deformation apart from axial strain. The conditions for ossification of tendons may well be realized within the muscular bellies, where pinnate muscle fibers are attached to the tendons as in *Meleagris* and *Grus* ([63]; Preuschoft, personal observation). Therefore, the length of an ossified tendon provides only information about the maximal distance between insertion and muscle, but not about the minimum distance, or the location of the muscle itself. This observation is in contrast to [5] as well as [24], who assume ossification alone in the free part of the tendons, outside, or distal to the muscle belly. In most galliform birds [63], [64], the ossified parts of long tendons parallel the long bones especially of the hind limbs and alternate with fibrous parts near the joints (where the point of deflecting the tendon changes).

The morphology of sauropod tail skeletons is similar to that in crocodiles. These amphibious archosaurs use their tails for propulsion in water, and their tail shape is clearly adapted to the external forces that are required by this function. In the case of sauropods, tail function is unknown, and an aquatic lifestyle is not seriously considered. The above-noted opinion that tails just counterbalance neck weight does not seem satisfying, because it implies that sauropods and many other dinosaurs were carrying considerable dead weight, or ballast, which is in clear contrast to the light-weight-constructions which have evolved in other parts of the body. If the effects of body weight alone are considered, sauropod tails in fact have adapted shapes, although not all details (like the divergent shapes of hemapophyses) can be explained.

### Mammals: Bending of the Neck under Weight

In mammals, necks are not really long in comparison to sauropods [5], but moderately “long” necks can be observed primarily among the large, cursorial, hooved mammals. Cattle, horses, and some cervids keep their seven-segment-necks often in a nearly horizontal posture. In most cervids, antilopes, and camelids, the posterior segments of the necks are kept horizontal, whereas the anterior segments approach the vertical. According to Christian and Dzemski [20], these necks are kept while resting at angles of about 40°–60° against the horizontal, and during locomotion at angles of 20°– 40°. At rest, the bending moments are reduced by lifting the necks, because the lever arms of segment weights follow the cosine of the angle of elevation. The elastic ligaments seem to be stretched to such an extent that they produce enough force, to keep the neck in balance. In locomotion, the mass moments of inertia of the body stem are increased by lowering the neck, which facilitates movements of the limbs against the trunk (Yamazaki pers. comm.; [34], [35], [36]).

The necks of cursorial herbivorous mammals can be classified as morphologically specialized for sustaining high bending moments in lateral view. In so far, mammalian necks and the tails of sauropods are similar. The farther removed the tension-resistant fibrous (nuchal ligaments) or muscles from the neck vertebrae, the longer are the force arms and the greater their torques. The lever arms are long because the nuchal ligaments and the muscles have their proximal insertions at the anterior thoracal vertebrae, (between the transverse processes and the ribs) and at the tips of the spinal processes. Especially the latter are elongated in the anterior thoracal segments, forming the “withers” of cursorial mammals (Fig. 6). The lack of bony outgrowth (like spinal processes or cervical ribs) on the cervical vertebrae yields freedom of mobility – but may require higher forces.

The neck construction of mammals also provides oblique muscles to sustain torsion, but these muscles usually bridge a large part or even the whole distance between thorax and the heavy head that, because of its mass, causes particularly high torsional moments (m. sternocleidomastoideus or m. brachiocephalicus; mm. splenius cervicis et capitis, m. semispinalis capitis in addition to the longus system). It should be noted that the necks of, e.g., carnivores do not have the high and narrow neck profile so typical of the large herbivores. The more circular cross sections of carnivores give their necks higher resistance against torsion. Because the necks of the hooved mammals are specialized for sustaining high bending moments, their neck construction permits the use of horns, antlers, or simply the frontal bone in intraspecific or interspecific fights (bovids, cervids, and giraffes). In terms of mechanics, these external forces are concentrated on the head and therefore have the longest possible lever arms along the neck, which is strongly bent when transmitting the external forces to the trunk. The fighting animals take care to keep these forces more or less in the sagittal plane and thus keep torsion within narrow limits. The energetically cheapest means to control bending of the neck under the influence of ventrally flexing moments, while keeping it in various positions, are long lever arms of muscles. The latter are obviously powerful, especially in males.

The mobility of necks in the large cursorial mammals is limited; they can usually reach their hindquarters by the teeth, but often not their backs. Extreme mobility of the neck can be observed among carnivorous mammals, especially in seals and elephant seals. These necks are characterized by strong vertebrae without long bony processes, and the very strong musculature is arranged in accordance to the muscles in other mammals. It should be noted, that necks of seals are – on land, under the influence of earth gravity – rarely kept in horizontal postures without bracing their heads on the ground. By contrast, seals show a clear tendency to place their necks into a vertical position for resting, basking, or display behaviors. If submerged in water, the necks lose their weight (but not their mass!) and therefore can be kept parallel to the body axis.

The tails of most mammals are unimportant for the general equilibrium and do not play a large role, because they neither contain much mass, nor are they kept in a more or less horizontal position in which they exert influence on the system. Some exceptions from this rule can be found among marsupials.

### Birds

Long-necked birds (ostriches, swans, geese, phasianids) can be seen as functional analogues of sauropods (see also [5]). During rest, slow walking and swimming, these birds regularly keep their necks upright, so that bending moments under the influence of gravity are minimized. Even in feeding, the lengths of necks are kept low by sigmoid curvatures (Fig. 2; [37]).

The usefulness of this analog, however, is doubtful, simply because the equipment with muscles theoretically can be, and in fact seems to be, quite different in both groups, in spite of the otherwise far-reaching homology of the musculature. In birds, the most obvious flexor of the neck is the m. longus colli ventralis [64], [65]. In some species, its tendons are ossified (crane and turkey, but not in storks nor in herons (personal observations; [65]). The clearly separated tendons diverge craniolaterally and reach separate insertions at the transverse processes of each vertebra. In the posterior direction, the muscles of both sides converge towards the crista ventralis of the thoracal vertebrae. This is not the direction of the ossified tendons in sauropod necks. The complete muscles of both sides clearly allow rapid protrusion of the head, while all cervical vertebrae are precisely controlled. This is vital for catching fast prey or in pecking, for example. In crocodiles, the m. longus colli ventralis is present, but not strongly developed. Functionally it is replaced by the m. costocervicalis (m. scalenus of [45]).

However, the subdivisions of the avian m. longus colli ventralis can hardly be considered as homologous to sauropod cervical ribs, because their common origin is along the ventral midline of the neck, instead of deviating from the midline, and because the tendons do not insert into the long and slender “processus costales” of the bird vertebrae. Instead, these structures are the insertions of the segmental m. longus colli lateralis [65]. In this point we disagree with Taylor and Wedel [5], who argue for the m. longissimus colli.

All we know about animal behavior indicates that every possible attempt is made to reduce the expenditure of muscle force; that is,horizontal neck posture is not probable as a frequently assumed or “resting” posture. The influence of gravity on neck posture can easily be observed in many birds (Fig. 3). If a long neck is kept upright in a resting position, its center of mass may well be located behind the vertical through the neck base (Fig. 11). In this case, muscles on both sides must exert tensile force to keep the intervertebral joints in balance. Such a posture obviously requires much less energy than a horizontal posture of the long neck (Fig. 11). Active ventral flexion by muscle activity is required if the head and neck are accelerated forward. In rapid movements, ventral flexion must be induced by muscle activity to overcome mass inertia of the neck. Acceleration of the neck and head for rapid ventral flexion of the neck takes place commonly in birds during capturing prey, pecking (woodpeckers, herons for example), and all similar activities, but it does not seem probable in the case of the herbivorous, browsing sauropods. Admittedly, slower flexion of the neck is and was initiated by weight – even if the neck was kept in a more or less upright posture. Under static conditions, a slight active ventral flexion by muscle activity is only required if the neck at rest is fully erect or inclined dorsally. The only task left in sauropods for the muscle and the ossified tendons is unilateral activity in order to keep the neck in balance against torsional moments.

Superficially, snakes seem to move their most cranial parts in a way similar to that postulated here for sauropods. These anterior parts of snake bodies, however are not necks, but anterior parts of the trunk, with its common equipment: Ribs and intercostal musculature. These anatomical elements have been identified by Preuschoft et al. [66] as torsion-resisting structures, but they belong to the trunk, not to the neck.

## Conclusions

Aside from the often-discussed bending in side view, necks of sauropods are exposed to torsion. This requires particular adaptations, especially because of the concentration of internal forces derived from torsion near the periphery of the twisted element. Very similar adaptations to torsional strength can be seen in crocodiles, which expose their admittedly short necks to huge torsional moments in the death roll. By contrast, the tails of sauropods do not show pronounced adaptation to torsion, and seem to have been carried more or less in a horizontal posture. In this respect, sauropod tails resemble the necks of large cursorial, herbivorous mammals. The high number of short neck segments is an indicator of neck flexibility, while long segments limit flexion, as do long dorsal and ventral apophyses.

The cervical ribs of some sauropods resemble functionally the tendons of a muscle group named in birds the m. longus colli ventralis, which gives raise to long tendons, inserting into each neck vertebra. The muscle bellies, however, are located more medially on the centra of the posterior vertebrae and do not insert into the processus costalis of the avian neck vertebrae. The direction of the cervical ribs in sauropods indicates a more lateral insertion, like that of the m. scalenus in crocodiles, which is contrary to Taylor and Wedel [5] who argue for the m. longissimus colli ventralis.

If acting on both sides, these muscles flex the neck ventrally – a movement that seems completely unnecessary in the heavy necks of sauropods if carried forwardly inclined. The existence of a strong ventral muscle is reasonable only if the neck is kept upright – a posture that saves energy. According to Christian [this collection], the m. longus colli ventralis may have an important function to counteract passive movements of the long sauropod neck in locomotion. No doubt, the muscle of which the ossified tendons seem to be the cervical ribs is perfectly suited to keep the neck in balance against torsional moments by unilateral activity. The forces produced by these muscles are further transmitted from the transverse processes to the spinal processes by the deep fibers of the m. longissimus system (multifidus cervicis) and m. splenius capitis.

In conclusion, the necks of diplodocids seem to have been very flexible, permitting smooth adaptation to a variety of postures, while those of brachiosaurids were more restricted and still more so the necks of mamenchisaurids. Unilateral activation of the m. longus colli ventralis or the mm. scaleni contributes in sauropods to shaking the head and twisting the neck, as well as to resisting torsional stresses in crocodiles.

## References

[1] Klein N, Remes K, Gee CT, Sander PM (2011) Biology of the sauropod dinosaurs: understanding the life of giants. Life of the Past (series ed. Farlow, J.) Bloomington: Indiana University Press. 344 pp.

[2] Sander PM, Christian A, Gee CT (2009) Response to sauropods kept their heads down. Science 323: 1671–1672. 19325098 10.1126/science.323.5922.1671

[3] Sander PM, Christian A, Clauss M, Fechner R, Gee CT, et al (2010) Biology of the sauropod dinosaurs: the evolution of gigantism. Biol Rev 86: 117–155. 10.1111/j.1469-185X.2010.00137.x PMC3045712 21251189

[4] Taylor MP, Naish D (2007) An unusual new neosauropod dinosaur from the Lower Cretaceous Hastings Beds Group of East Sussex, England. *Palaeontology* 50: 1547–1564.

[5] Taylor MP, Wedel MJ (2013) Why sauropods had long necks; and why giraffes have short necks. PeerJ 1:e36; 10.7717/peerj.36 PMC3628838 23638372

[6] Upchurch P, Barrett PM, Dodson P (2004) Sauropoda. In: Weishampel DB, Dodson P, Osmolska H, eds. The Dinosauria. Berkeley: University of California Press, pp. 259–322.

[7] Christian A (2010) Some sauropods raised their necks: evidence for high browsing in Euhelopus zdanskyi. Biol Lett 6: 823–825 10.1098/rsbl.2010.0359). 20519198 PMC3001369

[8] Ruxton GD, Wilkinson DM (2011) The energetics of low-browsing in sauropods. Biol Lett 7: 779–781. 21429913 10.1098/rsbl.2011.0116 PMC3169045

[9] Preuschoft H, Hohn B, Stoinski St, Witzel U (2011) Why so huge? Biomechanical reasons for the acquisition of large size in sauropod and theropod dinosaurs. In: Klein N, Remes K, Gee CT, Sander PM, eds. Biology of the sauropod dinosaurs: understanding the life of giants. Bloomington: Indiana University Press. pp. 197–218.

[10] Shorrocks B (2009) The behaviour of reticulated giraffe in the Laikipia district of Kenya. Giraffa 3(1): 22–24.

[11] Taylor MP, Wedel MJ, Naish D (2009) Head and neck posture in sauropod dinosaurs inferred from extant animals. Acta Palaeontol Pol 54: 213–220.

[12] Christian A, Dzemski G (2011) Neck posture in sauropods. In: Klein N, Remes K, Gee CT, Sander PM, eds. Biology of the sauropod dinosaurs: understanding the life of giants. Bloomington: Indiana University Press. pp. 251–260.

[13] Stevens KA, Parrish MJ (2005) Digital reconstructions of sauropod dinosaurs and implications for feeding. In: Wilson JA, Curry Rogers K eds. The Sauropods: evolution and paleobiology. Berkeley, CA: University of California Press. pp. 178–200.

[14] Stevens KA, Parrish MJ (2005) Neck posture, dentition and feeding strategies in Jurassic sauropod dinosaurs. In: Tidwell V, Carpenter K eds. Thunder lizards: the sauropodomorph dinosaurs. Bloomington: Indiana University Press. pp. 212–232.

[15] Frey E, Martin J (1997) Long necks of sauropods. In: Currie PJ, Padian K eds. Encyclopedia of dinosaurs. California San Diego: Academic Press, pp. 406–409.

[16] Martin J, Martin-Rolland V, Frey E (1998) Not cranes or masts, but beams: the biomechanics of sauropod necks. Oryctos 1: 113–120.

[17] Christian A (2002) Neck posture and overall body design in sauropods. Mitt Mus Natkd Berl, Geowiss Reihe 5: 269–279.

[18] Dzemski G, Christian A (2007) Flexibility along the neck of the ostrich (*Struthio camelus*) and consequences for the reconstruction of dinosaurs with extreme neck length. J Morph 268: 701–714. 17514722 10.1002/jmor.10542

[19] Romer AS (1956) Osteology of reptiles. Chicago: University of Chicago Press.

[20] Christian A, Dzemski G (2007) Reconstruction of the cervical skeleton posture of *Brachiosaurus brancai* Janensch, 1914, by an analysis of the intervertebral stress along the neck and a comparison with the results of different approaches. Fossil Rec 10: 38–49.

[21] Christian A, Heinrich WD (1998) The neck posture of *Brachiosaurus brancai* . Mitt Mus Natkd Berl, Geowiss Reihe 1: 73–80.

[22] Wedel MJ, Cifelli RL, Sanders RK (2000) Osteology, paleobiology, and relationships of the sauropod dinosaur *Sauroposeidon*. . Acta Palaeontol Pol. 45: 343–388.

[23] Cerda IA (2009) Consideraciones sobre la histogénesis de las costillas cervicales en los dinosaurios saurópodos. Ameghiniana 46: 193–198.

[24] Klein N, Christian A, Sander PM (2012) Histology shows that elongated neck ribs in sauropod dinosaurs are ossified tendons. Biol Lett: 10.1098/rsbl.2012.0778 PMC3497149 23034173

[25] Organ Ch, Adams J (2005) The histology of ossified tendons in dinosaurs. J Vert Paleontol 25: 602–613.

[26] Wolff J (1892) Das Gesetz der Transformation der Knochen. Berlin: Hirschwald.

[27] Pauwels F (1965) Gesammelte Abhandlungen zur funktionellen Anatomie des Bewegungsapparates. Berlin: Springer.

[28] Sverdlova NS, Witzel U (2010) Principles of determination and verification of muscle forces in the human musculaoskeletal system: Muscle forces to minimise bending stress. J Biomechanics 43: 387–396. 10.1016/j.jbiomech.2009.09.049 19880120

[29] Knese KH, Biermann H (1958) Die Knochenbildung an Sehnen und Bandansätzen im Bereich ursprünglich chondraler Apophysen. Zt f Zellfr 49: 142–187. 13625935

[30] Preuschoft H (1969) Statische Untersuchungen am Fuss der Primaten. I Phalangen und Metatarsalia. Z Anat Entw Gesch 129: 285–345. 4990262

[31] Preuschoft H (1970) Functional anatomy of the lower extremity. In: Bourne GH ed. The chimpanzee Vol 3. Basel: Karger-Verlag. pp. 221–294.

[32] Preuschoft H (1973) Functional anatomy of the upper extremity. In: Bourne G, ed. The chimpanzee, Vol 6. Basel: Karger-Verlag. pp.34–120.

[33] Kummer B (1959) Bauprinzipien des Säugerskeletts. Stuttgart: Georg-Thieme-Verlag.

[34] Witte H, Preuschoft H, Recknagel S (1991) Human body proportions explained on the basis of biomechanical principles. Zt Morphol Anthropol 78: 407–423. 1887666

[35] Preuschoft H, Witte H (1993) Die Körpergestalt des Menschen als Ergebnis biomechanischer Erfordernisse. In: Voland E ed. Evolution und Anpassung—Warum die Vergangenheit die Gegenwart erklärt. Stuttgart: Hirzel Verlag. pp 43–74.

[36] Preuschoft H, Witte H, Christian A, Recknagel S (1994) Körpergestalt und Lokomotion bei großen Säugetieren. Verhand dt Ges Zool 87: 147–163.

[37] Dzemski G (2006) Funktionsmorphologische Analysen langer Hälse bei rezenten terrestrischen Wirbeltieren zur Rekonstruktion der Stellung und Beweglichkeit langer Halse prähistorischer Tiere. Ph.D. Dissertation. Flensburg: University of Flensburg. 155p.

[38] Lehmann T (1974–77) Elemente der Mechanik, Band 1–3. Braunschweig: Vieweg & Sohn.

[39] [39] Dubbel H (1981) Taschenbuch für den Maschinenbau. Berlin: Springer. 1495p.

[40] Bock WJ, v. Wahlert G (1965) Adaptation and the form-function-complex. Evol 19: 269–299.

[41] Fürbringer M (1876) Zur vergleichenden Anatomie des Schultergürtels, 3. Teil, Morph Jb 1: 636–816.

[42] Preuschoft H (1976) Funktionelle Anpassung evoluierender Systeme. 5. Arbeitsgespräch zu Phylogenetik und Systematik, Lochmühle/Spessart. Aufsätze und Reden der Senckenberg. Naturforsch. Ges. Evoluierende Systeme I und II. Frankfurt: Kramer-Verlag. pp 98–117.

[43] Alexander RM (1985) Mechanics of posture and gait of some large dinosaurs. Zool J Linn So 83: 1–25.

[44] Walmsley CW, Smits PD, Quayle MR, McCurry MR, Richards HS, et al (2013) Why the long face? The mechanics of mandibular symphysis proportions in crocodiles. PLoS ONE 8(1): e53873 10.1371/journal.pone.0053873 23342027 PMC3547052

[45] Nishi S (1938) Muskeln des Rumpfes. In: Bolk L, Göppert E, Kallius E, Lubosch W, eds. Handbuch der vergleichenden Anatomie der Wirbeltiere. Berlin: Urban & Schwarzenberg. pp 351–446.

[46] Hohn B (2011) Walking with the shoulder of giants: biomechanical conditions in the tetrapod shoulder girdle as a basis for sauropod shoulder reconstruction. In: Klein N, Remes K, Gee CT, Sander PM, eds. Biology of the sauropod dinosaurs: understanding the life of giants. Bloomington: Indiana University Press. pp 182–196.

[47] Wedel MJ, Sanders RK (2002) Osteological correlates of cervical musculature in Aves and Sauropoda (Dinosauria: Saurischia), with comments on the cervical ribs of *Apatosaurus* . PaleoBios 22: 1–6.

[48] Gatesy SM (1990) Caudofemoral musculature and the evolution of theropod locomotion. Paleobiology 16(2): 170–186.

[49] Mallison H, Pfretzschner HU (2005) Walking with sauropods: modeling dinosaur locomotion in MSC visual NASTRAN 4D. J Vert Pal 25: 88A.

[50] Mallison H (2011) Rearing giants. Kinetic-dynamic modeling of sauropod bipedal and tripedal poses. In: Klein N, Remes K, Gee CT, Sander PM, eds. Biology of the sauropod dinosaurs: understanding the life of giants. Bloomington: Indiana University Press, pp. 237–250.

[51] Guibé J (1970) La musculature. In: Tome XIV, Fasc Grassé PP, editor. Traité de Zoologie. Paris: Masson. II: 701–812.

[52] Gasc JP (1981) Axial musculature. In: Gans C, Parsons TS (eds.). Biology of the Reptilia. London: Academic Press Vol. 11, pp. 355–435.

[53] Preuschoft H, Reif WE, Muller WH (1974) Funktionsanpassungen in Form und Struktur an Haifischzahnen. Zt Anat Entwlgs 143: 315–344. 4418599

[54] Willey JS, Biknevicius AR, Reilly SM, Earls KD (2003) The tale of the tail: limb function and locomotor mechanics in *Alligator mississippiensis.* . J Expl Biol 207: 553–563. 10.1242/jeb.00774 14691103

[55] Fechner R (2009) Morphofunctional Evolution of the pelvic girdle and hind limb of Dinosauromorpha on the lineage to Sauropoda. Ph.D. Dissertation. München: University of München. 211p. Online available: http://edoc.ub.unimuenchen.de/10954/1/Fechner_Regina.pdf

[56] Fechner R, Stratmann M, Gößling R, Sverdlova N (2013) The functional role of the ischiopubic membrane for the mechanical loading of the pubis in the domestic fowl (*Gallus gallus*). J Anat 222: 305–312. 23171269 10.1111/joa.12015 PMC3582250

[57] Henderson DM (1999) Estimating the masses and centers of mass of extinct animals by 3-D mathematical slicing. Paleobiology 25: 88–106.

[58] Thulborn RA (1989) The gaits of dinosaurs. In: Gillette DD, Lockley MG eds. Dinosaur tracks and traces. Cambridge: Cambridge University Press. pp.39–50.

[59] Bird RT (1944) Did *Brontosaurus* ever walk on land? Nat Hist 53: 60–67.

[60] Alexander RM (1989) Dynamics of dinosaurs and other extinct giants. New York: Columbia University Press. 167p.

[61] Preuschoft H, Gudo M (2005) Die Schultergürtel von Wirbeltieren: Biomechanische Überlegungen zu den Bauprinzipien des Wirbeltierkörpers und zur Fortbewegung von Tetrapoden. Zblt Geo Pal, Teil II 2005: 339–361.

[62] Wedel MJ (2006) Origin of postcranial skeletal pneumaticity in dinosaurs. Integrative Zoology 2: 80. 10.1111/j.1749-4877.2006.00019.x 21395998

[63] Blickhan R (2007) Biomechanik der axialen aquatischen und der pedalen terrestrischen Lokomotion. Aachen: Shaker Verlag. p 179.

[64] Nickel R, Schummer A, Seiferle E (1973) *Lehrbuch der Anatomie der Haustiere, Band 5 Anatomie der Vögel.* Stuttgart: Medizinischer Verlag.

[65] Bock WJ (1974) The avian skeletomuscular system. In: Farner DS, King JR, Parkes KC (eds.). Avian Biology Vol. 4. New York: Academic Press, pp. 119–257.

[66] Preuschoft H, Schulte D, Distler C, Witzel U, Hohn B (2007) Body shape and locomotion in monitor lizards. Mertensiella 16: 58–79.

[67] Abel O (1909) Die Rekonstruktion des *Diplodocus* . Abh zool-bot Ges Wien 5: 1–60.

